# Supplementary material for: Potential Transformation of Contaminated Areas into Public Parks: Evidence from São Paulo, Brazil
Source: Int J Environ Res Public Health. 2022 Sep 21;19(19):11933. doi: 10.3390/ijerph191911933 (PMC9564805; doi:10.3390/ijerph191911933)
Supplement: Supplementary file 1 [file ijerph-19-11933-s001.zip › ijerph-1878277-supplementary.pdf]

Table S1. History and Actions carried out in the areas of case studies.

Park	Year	Situation
**Jardim Primavera**	1950	Unoccupied area
	1968	Beginning of sand mining
	1979<br>1979	Start of the dump<br>End of sand mining
	1981<br>1981	Complaint by the population to the Subprefecture<br>Grounding of the smaller pit
	1983	Preparation of the Jacuí Landfill project<br>Transformation into controlled landfill<br>Installation of drains
	1988	CETESB Tatuapé was contacted- Accident in a residence<br>Closure of the controlled landfill
	1990	Park pilot project - SVMA/DEPAVE
	1991-1992	The construction of the park is initiated<br>Vegetal recomposition<br>Construction is interrupted
	1994-1998	Gas migration to homes<br>CETESB Technical Inspections
	1995	Planting of eucalyptus trees by SVMA
	2001	Significant gas finding- COMGÁS<br>Improper operation of drains

Park	Year	Situation
**Villa Lobos**	1950	Unoccupied area
	1958	Beginning of occupation, undergrowth, land movements<br>Boaçava Stream crossed the area
	1968	Earth movement in ditches<br>Roads used for sand extraction
	1974	Arrangement of dredged material in the South section of the area<br>Vegetal recomposition in a pit<br>New land movement in the Northeast part
	1977	Pit grounded in the Northwest part<br>Ferries with dredged material were found
	1980	Architect Decio Tozzi identifies degraded area<br>Development of the project for its recovery
	1986	Appearance of new paths formed within the area<br>Residential occupation with soccer field.
	1986	Western Part - CEAGESP residue<br>East- dredged material from Pinheiros River<br>Central part- construction waste deposit
	1987	Project of a Contemporary Theme Park
	1988	Camargo Correia bidding - Responsible for the construction of the park<br>State Decrees No. 28.335/1988 and No. 28.336/1988- Area declared as a public utility for expropriation
	1989	Beginning of the implementation of Villa-Lobos State Park<br>Boaçava Stream channeled<br>Removal of families living on site
	1990	Tree Planting- 50,000 trees/ 300 species
	1994	Park partially built, different from the original design

	2002	Law 13.302- Creates the Municipal Park Jardim Primavera		2004	Transfer of park administration to SIMA State Decree No. 48.441/2004<br>On-site maintenance problems - adaptation of species diversity<br>Development of executive projects for the park expansion<br>Resolution SMA No. 20, of March 7, 2004- creates the Villa-Lobos State Park Orientation Council
	2004	CETESB calls for detailed study in the area		2006	Park completed with 24,000 trees planted<br>Environmental entities - Public Civil Action against the State with the participation of the Public Prosecutor's Office
	2007-2008	Preliminary and confirmatory assessment		2007-2008	Preliminary and confirmatory assessment
	2008	The implementation of the park is resumed- Phase I<br>Construction is interrupted		2008	Planting of over 800 seedlings in the area
	2010	Construction is resumed- Phase I		2009	Confirmatory evaluation - External area, surroundings of the park and Marginal Tietê
	2011	Construction is finished- Phase I<br>Development of Reference Term - Phase II		2009	Park received 8,404 native seedlings<br>Inauguration of the following spaces: environmental village, administration and headquarters of the Military Police and Samu
	2012	Services hiring- Installation of vapor extraction system - economic unfeasibility		2010	Inauguration of the following spaces: Ouvillas, the Ruth Cardoso Orchid Greenhouse
	2012	Interdiction of the area - Public Prosecutor's Office		2012	Detailed assessment, risk assessment and intervention plan
	2013	Gas monitoring proposed by SVMA		2013	Environmental education Center- Villa Lobos Library
	2014-2016	Detailed assessment, risk assessment and intervention plan<br>Gas monitoring in the area - Methane and VOCs		2020	The construction of the park is concluded, and it’s considered as successful case in Brazil, especially in the city of São Paulo.
	Feb/2017	SVMA requested CETESB to issue the term of area rehabilitation for declared use.			
	May/2017	CETESB approved the intervention plan for reuse of the area.			

		Technical opinion favorable for the partial reopening of phase I of the Park.			
	Dec/2018	Inspection of the investigation made by the Public Prosecutor's Office in the area.			
	2020	32 years after the closure of the controlled landfill, the park was not yet concluded for the benefit of the population.			

References on the text. Prepared by the author.
